# The epidemiology of airplane headache: A cross-sectional study on point prevalence and characteristics in 50,000 travelers

**DOI:** 10.1177/03331024221092408

**Published:** 2022-04-13

**Authors:** Fabian Konrad, Andreas Moritz, Michael Moritz, Johann Georg Keunecke, Felix Tischler, Johannes Prottengeier

**Affiliations:** 1 Faculty of Medicine, Friedrich-Alexander University Erlangen-Nuremberg (FAU), Erlangen, Germany; 2 Department of Anesthesiology, University Hospital Erlangen, Erlangen, Germany; 3 Department of Anesthesiology, Kepler University Hospital, Linz, Austria

**Keywords:** Airplane headache, barotrauma, cross-sectional data, headache disorders, primary, headache attributed to airplane travel, headache attributed to disorders of homeostasis, International Classification of Headache Disorders, secondary headache disorders

## Abstract

**Background:**

The current knowledge on the epidemiology and clinical manifestation of airplane headache is mostly derived from case series and small cohort studies without evidence from large populations.

**Methods:**

This cross-sectional study was conducted over a five-month period in the arrival area of two international airports in Germany. 50,000 disembarking passengers were addressed about headaches during their flight to determine headache prevalence, and those confirming and willing to participate underwent a structured interview.

**Results:**

Headache during travel was reported by 374 passengers (0.75%), and 301 underwent a structured interview. One hundred and one (0.2%) met the diagnostic criteria of airplane headache. Six passengers suffered from migraines and 134 from tension-type headaches. The differences in the age and gender distribution between the airplane headache and non-airplane headache groups were not statistically significant. The onset (79.2%), duration (82.2%), and location (73.3%) of airplane headache mostly complied with current diagnostic criteria but pain intensity (42.6%) and quality (42.6%) did less so.

**Conclusion:**

Our data suggest a substantially lower prevalence of airplane headaches than previously reported. The pain intensity and quality seem less characteristic than assumed, suggesting a need to refine the current diagnostic criteria.

## Introduction

Since the beginning of aviation, headaches during flights have been frequently reported. For a long time, they were solely attributed to barotrauma or respiratory infection (1). Since the first description of “headache associated with airplane travel” in the literature in 2004 (2), this condition has gained increasing attention from the scientific community, culminating in its recognition as a distinct type of secondary headache (“headache attributed to a disorder of homoeostasis”) in the third International Classification of Headache Disorders (ICHD-3) in 2013 (2–5). In this classification it is described as an often severe headache, usually unilateral and periocular and without autonomic symptoms, occurring during and caused by aeroplane travel (4,5). Initially, our medical knowledge on the epidemiology, clinical features, diagnostic criteria, and possible treatment options was derived from individual case reports and small case series (6–9). This changed after the publication of the results of a case series by Mainardi et al., (10,11) when airplane headache (AH) was recognized as a distinct headache disorder, thus opening up opportunities for further structured research.

The small number of earlier published cases suggested that AH might be a rare condition. However, a cohort study by Potasman et al. (12) found that more than 5% of travelers attending a travel clinic for a large spectrum of other complaints were affected by AH, indicating that it might not be rare at all. More recent cohort studies by Mainardi et al. (13) Bui et al. (14) and Lima et al. (15) observed similar prevalences between 4% and 14.7% in significantly preselected populations. Therefore, it must be postulated that the true prevalence of AH remains unknown up to date, as no studies are available that have investigated fully representative population samples. As there are more than 3.5 billion airline passengers each year, investigations into the true prevalence of AH are indisputably needed (16).

Several studies described severe or even excruciating pain as one of the distinct characteristics of AH, with mean scores of up to 9.6 on the verbal numerical rating scale (VNRS), leading to its inclusion in the current ICHD-3 criteria (3-6,7,10,11,13). However, other studies reported lower intensities, but this is not reflected in the ICHD-3 (3-5,12,14,17).

Consequently, we considered it necessary to investigate the epidemiology of headaches associated with airplane travel further to understand their clinical significance better and gain more detailed insights into their characteristics.

In this investigation, we aimed to collect basic epidemiological data on AH and to describe its clinical manifestation based on a large data set. This is the first study to evaluate an unselected sample of passengers immediately after their arrival at two international airports.

## Methods

### Study objectives, design, and population

This cross-sectional study‘s primary objective was to evaluate the point prevalence of AH in a large population of passengers. The secondary objectives included refining data on the clinical appearance of AH and determining the prevalence of other headache types during air travel.

Each individual arriving by airplane was eligible for participation in the study.

The study took place over a five-month period from November 2013 to March 2014 at the airports of Nuremberg and Munich in Germany that together serve more than 40 million international passengers per year (18).

The recruitment was conducted in the immediate arrival area, where every passenger had to pass by the survey desk to exit. Our investigators approached all debarking passengers one-on-one and addressed them about headaches during the flight. Those identifying themselves as experiencing AH and willing to participate in our study would then proceed to our interview booth, where they went through a structured interview.

We also documented the number of travelers who indicated that they had suffered from an acute headache but were unwilling to participate in our study. Considering the resources available to perform this study, we had predetermined a number of 50 000 passengers to be addressed, whom we would ask whether they had experienced headaches during the flight.

### Structured interview

The questions of our structured interview were the result of discussions amongst our department’s pain experts using the pain center’s headache assessment sheet, combined with physiological and logistical considerations of airplane travel, including the diagnostic criteria for AH described by the International Headache Society as well as general epidemiological data (3-5).

Based on the responses, we distinguished an AH group and a non-AH group and assigned participants accordingly to either group. Crucial for the differentiation between the different headache entities were only the headache symptoms experienced on the particular flight from which our participants were departing.

### Statistical analysis

Statistical analysis was performed using IBM SPSS Statistics for Windows, version 26.0 (IBM Corporation, Armonk, NY, USA). Categorical data were presented as frequencies and percentages (%). Continuous data, such as age, weight, height, and travel time, were presented as the mean ± standard deviation or as the median plus the interquartile range. Differences in height, weight, and age between the AH and non-AH groups were examined using the Kruskal-Wallis test. The sex distribution was determined using the odds ratio. The level of statistical significance was set at 0.05, and 95% confidence intervals (CI) were calculated as appropriate.

## Results

Out of 50 000 passengers addressed, 374 (0.75%) reported headache episodes during their flight. 19.5% (73/374) of those who had experienced headaches were unwilling to be interviewed and could not be analysed in more detail. Consequently, 301 participants were interviewed to the full extent. Based on the ICHD-3 criteria, 175/301 (58.13%) participants were identified as AH (4,5). Among these, five simultaneously met the ICHD-3 criteria for migraine without aura (MO) and 69 those for tension-type headache (TTH). These participants were not counted as AH but as primary headache, leaving a total of 101/301 (33.55%) participants fulfilling the pre-specified AH criteria. We found a point prevalence of headache during airplane travel of 0,75% (374/50,000), for AH of 0.35% (301/50,000) and strictly AH of 0.2% (101/50,000). These 101 participants will be referred to as the AH group and further described, whereas the other 200 interviewed will be referred as the non-AH group.

Distributions for age (p = 0.189), height (p = 0.778), and weight (p = 0.734) in the two study groups did not show statistically significant differences in the Kruskal-Wallis test. The odds ratio for gender distribution did not show a significant difference (OR = 0.717, 95% CI = 0.442, 1.163) between the two groups. Details are presented in Tables 1 and 2.

**Table 1.** Gender distribution in passengers with airplane headache (n = 101) and non-airplane headache (n = 200).

	Females	Males	No data	Total
AH, n (%)	45 (15)	56 (18.6)	–	101 (33.6)
Non-AH, n (%)	102 (51)	91 (45.5)	7 (3,5)	200 (76.4)
Total, n (%)	147 (48.8)	147 (48.8)	7 (2.3)	301 (100)

AH, airplane headache.

**Table 2.** Age, weight, and height of passengers with airplane and non-airplane headaches.

	Age (y)	Weight (kg)	Height (cm)
AH			
mean (SD)	41.81 (14.65)	73.1 (14.42)	172.83 (9.38)
95% CI	39, 44.88	70.3, 76.11	171.13, 174.91
median (Q_1_, Q_3_)	41 (31, 55)	73 (64, 81)	172 (165, 179.5)
Non-AH			
mean (SD)	44.16 (13.27)	74.25 (16.41)	173.31 (9.76)
95% CI	42.24, 46	71.93, 76.57	171.9, 174,76
median (Q_1_, Q_3_)	45 (33, 54)	73.5 (60.25, 85)	173 (165.5, 180)

AH, airplane headache; CI, confidence interval; Q, quartile; SD, standard deviation.

Six (2%) and 134 (44.52%) of 301 participants were classified as MO and TTH, respectively. No participant was classified as having cluster headaches or trigeminal neuralgia.

During the study period, no passengers were treated for headaches by medical services at the Munich or Nuremberg airport.

## Airplane headache

### Clinical appearance of AH

The clinical features of AH as per the ICHD-3 criteria in our participants are listed in Table 3 (4,5). Eighty participants (79.2%) described the onset occurring during phases of the flight with pressure changes, whereas 14 (13.9%) described the onset at cruising altitude. AH episodes mostly ended once stable cabin pressure conditions were reached (n = 24, 23.8%) or during (n = 19, 18.8%) and after disembarking (n = 4, 4%). In 14 (13.9%) participants, the pain persisted and was still present when completing the survey. In some cases, the pain ended during the descent (n = 8, 7.9%) or landing (n = 21, 20.8%). Eighty-four (83.2%) participants described one AH episode only, 11 (10.9%) described two, and 5 (5%) described more than two episodes. In most cases, a sudden beginning (n = 63, 62.4%) without premonition (n = 77, 76.2%) was described. Almost all AHs occurred during physical rest (n = 95, 94.1%). The AH episodes had a sudden ending in almost half of the cases (n = 44, 43.6%). The mean severity of pain was 5.7 ± 2.3 on the VNRS. A wide variety of pain qualities were observed in AH (Table 4). Strictly unilateral pain was only described by 21 (20.8%) participants (Table 3). In addition to the typical locations of AH, temporal (n = 35, 34.7%) and occipital (n = 18, 17.8%) locations were common. Some participants described facial (n = 7, 6.9%) or parietal (n = 10; 9.9%) pain. Participants described pain radiating to the orbital area (n = 37, 35.9%), ears (n = 5, 5%), entire face (n = 12, 11.9%), and neck (n = 11, 10.9%).

**Table 3.** International Classification of Headache Disorders-3 criteria for headache attributed to airplane travel.

Headache	n	%
During airplane flight	101	10
Onset during phases with pressure change	80	79.2
ascent	17	16.8
take off	11	10.9
descent	30	29.7
landing	22	21.8
Duration ≤30 min	83	82.2
≤10 min	34	33.7
>10 min and ≤30 min	49	48.5
>30 min	18	17.8
Severe intensity (7–10)	43	42.6
Mild/moderate (1–6)	58	57.4
Location	74	73.3
unilateral	21	20.8
orbital	36	35.6
frontal	54	53.5
parietal	10	9.9
Quality	43	42.6
stabbing	19	18.8
pulsating	27	26.7
Sinus disorder	0	0

Note: For the parameters location and quality multiple answers were possible. Intensity rated in verbal numerical rating scale.

**Table 4.** Pain quality of airplane headache.

	n	%
Pressing	40	39.6
Throbbing	27	26.7
Stabbing	19	18.8
Blunt	13	12.9
Knocking	9	8.9
Hammering	7	6.9
Sharp	7	6.9
Pulling	7	6.9
Cutting	5	5
Drilling	5	5
Flashing	3	3
Burning	4	4
Tearing	4	4
Spasmic	3	3

Note: Multiple answers were possible.

### Flight duration and frequency of AH

Seventy-three participants (72.6%) were occasional flyers (1–10 times a year). The mean travel time of participants with AH was 5.14 ± 3.25 hours (range 1–13).

Almost half of participants with AH (n = 47, 46.5%) reported regular headache attacks during flights. Twenty-one participants (20.8%) reported their first AH episode. Occasional episodes occurred in 28 (27.7%) participants. Five (5%) participants reported episodes on every flight. The majority of participants described their AH episodes (n = 61, 60.4%) to be similar in quality. Only five participants (5%) reported a consultation with a medical professional for AH.

Most of the participants denied having previously diagnosed primary headaches (n = 79, 78.2%). Pre-existing MO was described by eight (7.9%), TTH by nine (8.9%), and cluster headache by three (3%) participants. Eight participants (7.9%) reported having received medical treatment for headaches in general at some point.

### Accompanying symptoms in AH

Accompanying symptoms were described by almost half of the participants (Table 5). Fourteen participants (13.9%) described the beginning of accompanying symptoms simultaneously to the beginning of the headache, seventeen (16.8%) during the headache, seven (6.9%) before the headache, and two (2%) after the headache.

**Table 5.** Accompanying symptoms of airplane headache.

	n	%
Nausea	25	24.8
Clogged nose	10	9.9
Dizziness	5	5
Fatigue	4	4
Dry mouth	3	3
Clogged ears	3	3
Unsteadiness	2	2
Restlessness	2	2
Lacrimation	2	2
Stomach ache	2	2
Photophobia	1	1
Tinnitus	1	1
Vomiting	1	1
Shivering	1	1
Irritability	1	1
Circulatory problems	1	1
Total	48	47.5

Note: Multiple answers were possible.

### Self-medication, self-invented maneuvers, and prevention

Thirty-six participants (35.5%) reported self-medication, twenty (19.8%) used analgesics and nine (8.9%) sedatives. Forty-seven participants (46.5%) indicated that they would take precautions to prevent AH during their next flight. Twenty (19.8%) took analgesics, and 18 (17.8%) aimed at diligent hydration before or during the next flight. Improvement was accomplished through self-invented therapies, such as the Valsalva maneuver (n = 4, 4%), acupressure (n = 4, 4%), massage (n = 3, 3%), chewing gum (n = 9, 8.9%), physical activity (n = 2, 2%), or rest (n = 14, 13.9%). Among these, hydration (n = 8, 7.9%) and analgesics (n = 22, 21.8%) were considered the most helpful. Other participants described worsening of the headache after consumption of alcohol (n = 2, 2%), acupressure (n = 2, 1%), massage (n = 2, 2%), motion (n = 6; 5.9%), and hydration (n = 13, 12.9%). Five participants (5%) reported their AH to the cabin crew, and one participant required medical assistance during the flight.

### Upper respiratory tract disorders

Four participants (4%) described coexisting asthma and five pollinosis (5%). Previous injuries or surgical procedures of the nose or sinuses were reported by three participants (3%). No symptoms of acute sinusitis or a common cold were described. Five participants (5%) had previously diagnosed nasal septum deviation. Twenty-seven (26.8%) participants were smokers.

### Headache triggered by other homoeostasis disorders

Headache episodes during instances of pressure change unrelated to flying were reported by 44 participants (43.6%), 38 (37.6%) of whom experienced headache episodes with weather changes, nine (8.9%) during descending from mountain passes, nine (7.9%) during cable car rides, and three (3%) during skiing. Sixty-four (63.4%) individuals reported other symptoms during pressure changes such as nausea (n = 6, 5.9%), dizziness (n = 3, 3%), ear pain (n = 4, 4%), and body aches (n = 2, 2%).

## Non-AH headache

Basic epidemiological data for other specific headache types in the non-AH group are shown in Tables 6 and 7. Of 74 participants fulfilling the ICHD-3 diagnostic criteria for a primary headache (MO or TTH) as well as AH, 48 (67,6%) do not have known headache diagnoses.

**Table 6.** Gender distribution in passengers with non-airplane headache.

	Female	Male	No data	Total
MO	5 (2.5 %)	1 (0.5 %)	–	6 (3 %)
TTH	65 (32.5 %)	66 (33 %)	3 (1.5 %)	134 (67 %)
Unclass.	32 (16 %)	24 (12 %)	4 (2 %)	60 (30 %)
Total	102 (51 %)	91 (45.5 %)	7 (3.5 %)	200 (100 %)

MO, migraine without aura; TTH, tension type headache; Unclass., unclassified.

**Table 7.** Age, weight, and height in non-airplane headache.

	Age	Weight	Height
MO			
mean (SD)	44.67 (11.31)	77.33 (6.02)	172.17 (8.82)
95% CI	32.8, 56.53	71.01, 83.65	162.91, 181.42
median (Q_1_, Q_3_)	47 (32.25, 53.75)	77 (72.25, 83.25)	174 (166.5, 179.25)
TTH			
mean (SD)	44.67 (13.5)	74.97 (17.5)	174.22 (9.64)
95% CI	41.9, 46.59	71.71, 77.88	172.37, 175.77
median (Q_1_, Q_3_)	46 (33.5, 54)	74 (61, 86)	173 (167.75, 180)
Unclass.			
mean (SD)	42.93 (13.05)	72.25 (14.36)	171.32 (9.98)
95% CI	39.15, 46.1	68.41, 76.16	168.56, 173.94
median (Q_1_, Q_3_)	44 (33, 54)	70 (60, 82)	170 (164, 178)

CI, confidence interval; MO, migraine without aura; Q, quartile; SD, standard deviation; TTH, tension type headache; Unclass., unclassified.

## Unclassified headache

Sixty participants (19.9%) experienced headaches that did not correspond to any ICHD-3 type. The characteristics of these headaches are presented in Table 8.

**Table 8.** Characteristics of unclassified headache (n = 60).

	n	%
During flight	60	100
Onset during phases with pressure change	26	46.4
ascent	6	10.7
take off	6	10.7
descent	12	20
landing	2	2
Duration ≤30 min	31	55.4
Severe intensity (7–10)	17	30.3
Mild/moderate pain (1–6)	39	43.6
Location		
unilateral	3	5
orbital	8	13.3
frontal	30	50
parietal	4	6.7
temporal	22	36.7
Quality		
stabbing	6	10
pulsating	9	15
pressing	29	48.3
hammering	11	18.3
dull	15	25
Sinus disorders		
surgery	8	13.4
injury	2	3.3
Respiratory symptoms	37	68.5
rhinitis	9	15
sinusitis	10	16.7
cough	3	5
Pre-existing headache		
Migraine	13	21.7
Tension-type headache	6	10
Cluster headache	1	1.7

Note: For the parameters location and quality multiple answers were possible. Intensity rated on verbal numerical rating scale.

## Discussion

### Prevalence of headaches during air travel

Our study suggests that headaches of any kind are rare occurrences during airplane travel, with a combined prevalence of 0.75% (374/50,000). This frequency is considerably lower than what was previously described and is also lower compared to a variety of point-prevalence investigations for headache in the general population reporting frequencies of 2.98% to 14.6% (12,19–26). Furthermore, AH accounted for only approximately one-third (103/301) of the headache types reported, less than that reported in earlier investigations (12–15).

Our population selection method stands apart from previous studies. For example, Potasman et al. (12) and Mainardi et al. (13) interviewed individuals actively seeking medical attention. Bui et al. (14) advertised their questionnaire on headache patient organizations’ Facebook pages. Both recruitment strategies may present a “positive” selection bias as a preselected subpopulation of patients, not passengers, was addressed. This might have resulted in a false high prevalence.

Interestingly, among the 175 passengers who met the ICHD-3 criteria for AH, a large proportion (42.3%) also met the ICHD-3 criteria for primary headaches (4,5). This emphasizes the uncertainties underlying an AH diagnosis, likely caused by the many similarities in pain severity, location, and quality. If we want to differentiate between different headache types more precisely, it will be helpful to assess accompanying symptoms, such as nausea, photo- or phonophobia, or the effect of motion and posture. None of these are currently included in the diagnostic criteria for AH (4,5).

### Clinical profile

During the analysis of our data set, we encountered the practical problem that a large group of patients (60/301, 19.9%) could not be assigned to any of the predefined groups of headaches in the ICHD-3. Their symptoms mostly met the ICHD-3 criteria of AH, but the subjective description of their complaints—especially respiratory symptoms, pain location and quality, amongst others—fell outside of the ICHD-3 criteria and thus prohibited the inclusion of these participants in the AH group (4,5).

The main reason for not assigning participants to the AH group was the presence of subjective respiratory symptoms (32/301, 10.6%), which requires further explanation. In aerospace medicine, upper respiratory tract infection and sinusitis cause severe pain during flights (27). The American Society of Aerospace Medicine Specialists Guidelines recommend that aviators remain grounded when affected by upper respiratory tract infection or acute sinusitis because both can cause incapacitation during atmospheric perturbations, resulting in flight safety issues (28). Consequently, Mainardi et al. (10) proposed, and the current ICHD states, that acute sinusitis excludes the diagnosis of AH (4,5). This exclusion seems necessary to formally separate AH and barotraumatic sinusitis as the suspected underlying causes of AH have a similar pathomechanism as barotraumatic sinusitis and include barotrauma due to atmospheric pressure changes, local inflammation of the nasal mucosa, and anatomical variation of the sinuses (6,7,17).

### Proposal for amending the diagnostic criteria for airplane headache

AH can only be diagnosed if the criteria of the ICHD are met. However, the current ICHD-3 reflects mainly data derived from earlier case series and cohort studies, which may be subject to relevant selection bias, as explained above. Consequently, we believe that these diagnostic criteria are not truly representative of all patients suffering from this distinct disorder.

Per our results, several passengers suffering from headaches during air travel do not meet the long-established criteria of any primary headache type, but also cannot be classified as AH, even though the similarities are often striking. Classification is often hindered by sub-criteria that seem too strict and without pathophysiological substance.

Considering the vast inter-individual differences in both the quantity and quality of pain, we would like to initiate a discussion on the current ICHD-3 criteria for AH with the aim to widen the ICHD-3 criteria for AH to represent more accurately all individuals affected by this disorder.

Our reasoning is as follows:

1. Currently, there are no proven pathophysiological mechanisms or anatomical correlations for the development of AH. Any localization from the forehead to the occiput, left and right, and uni- and bilateral should be considered as compatible with AH.
2. Travelers suffering from AH may undergo only a few episodes and may never seek medical attention. Perhaps this inexperience with headaches may lead to imprecise formal descriptions of pain quality. While experience and precision may be expected from patients experiencing chronic pain, it is not necessary to demand them from the inexperienced. The criteria for the description of pain quality proposed by the International Headache Society may need to be widened accordingly.
3. Considering some previous case series and cohort studies and our findings, moderate pain seems far more common than assumed previously. We propose classifying headaches with typical timing and clinical appearance, but not of severe intensity, as AH.

## Limitations

Our study may be subject to a negative selection bias analogous to the “healthy worker effect”, resulting in a lower point prevalence in comparison to other point prevalence studies for headaches (19–26,29). In more detail, especially among patients suffering from severe forms of AH, the negative emotional impact may lead to a tendency to avoid airplane travel (10,11,13).

Consequently, our study may not only be subject to a negative selection bias with regards to AH prevalence, but also to an underestimation of pain severity. Furthermore, passengers suffering headaches might also not have been willing to disclose their complaints and might have been incorrectly counted as non-afflicted individuals.

Aiming for the point prevalence and investigating only a single headache episode, the currently available diagnostic criteria made it difficult to differentiate clearly between primary and secondary headaches in several cases because the symptoms used for classification made both diagnoses conceivable in that given moment. Per section D of the ICHD-3 diagnostic criteria of AH, we excluded those dual cases from our case count. However, due to the great number of overlapping cases, we would also encourage future studies that aim at providing additional pathophysiological ground to distinguish more accurately between those headache entities that may not be differentiated by their symptoms alone.

## Conclusions

In the present study, episodes of any type of headaches during airplane travel were far less frequent than based on the literature. Based on the resulted prevalence of AH in our study and with an estimation of 3,5 billion airline passengers per year, approximately 26 million travellers should suffer from such a condition. It seems possible that pain quality, location, and severity in AH are not as distinct as suggested beforehand. Consequently, we recommend a review of amendments to the AH diagnostic criteria in the ICHD.

## Article highlights

- In this large population of airline passengers, headaches during travel were infrequent occurrences. Distinct airplane headache is a less common condition than previously suggested.
- Airplane headache symptoms were less typical than described in the literature.
- We advocate the amendment of the ICHD-3 diagnostic criteria to accommodate all clinically compatible forms of airplane headache.
